# Skin Lesions and Systemic Reactions in Humans Infested by Blackflies (Diptera: Simullidae) in Recreational Areas in Southeastern Poland

**DOI:** 10.3390/jcm10040788

**Published:** 2021-02-16

**Authors:** Monika Sitarz, Alicja Buczek, Weronika Buczek

**Affiliations:** 1 Chair and Department of Conservative Dentistry with Endodontics, Faculty of Medical Dentistry, Medical University of Lublin, 20-093 Lublin, Poland; monika.sitarz@umlub.pl; 2 Chair and Department of Biology and Parasitology, Faculty of Health Sciences, Medical University of Lublin, 20-080 Lublin, Poland; wera1301@gmail.com

**Keywords:** *Simulium*, Simuliidae, blackfly bite, blackfly dermatitis, simuliosis, bloodsucking arthropods

## Abstract

Due to their mass occurrence in some environments and high aggressiveness, blackflies (*Simulium* spp.) represent the most bothersome arthropods attacking humans. In this study, we describe the medical effects of blackfly infestations in humans in southeastern Poland. Local and systemic reactions to blackfly bites were monitored in 418 patients (61.24% of females and 38.76% of males) of medical centers. Only skin lesions at the site of the bites were found in 88.52% of the patients, whereas accompanying systemic reactions were diagnosed in 11.48%. The most common signs observed in the area of the bites were pruritus (94.74%), burning (55.02%), edema (44.02%), and erythema (40.91%). The skin lesions, which were most often grouped small papules and papular and purpuric lesions with a varied range, typically persisted for several days, or for several weeks in some patients. Statistical analyses confirmed that the persistence of the skin lesions did not depend on the sex of the patients and the number of blackfly infestations. The systemic reactions to the components of the blackfly saliva were manifested by headache, increased body temperature, arthralgia, lymphadenopathy, and menstrual disorders in the females. The patients were most often attacked simultaneously by many blackflies on exposed parts of the body, mainly the upper limb, lower limb, head, and neck areas. The investigations indicate that blackflies are an important etiological factor of dermatitis and other symptoms in humans; hence, they should be considered in clinical diagnosis.

## 1. Introduction

Blackflies (Diptera: Simuliidae) are arthropods with a worldwide distribution [1]. Approximately 10–20% of species of over 2330 representatives of this family parasitize humans and animals, causing serious medical (e.g., [2,3,4,5,6]) and veterinary problems [7,8] and, consequently, large economic losses (e.g., [9,10,11,12]).

Blackflies (*Simulium* spp.) occur most abundantly in areas located near fast-flowing rivers and streams, which are habitats for their pre-imaginary stages, i.e., larvae and pupae. Females of most species of these insects released from pupae obligatorily ingest blood of vertebrates, which is indispensable for their development and oviposition [13,14,15].

Blackflies are important vectors of pathogens with considerable importance for public health, for instance, the best-known and widespread filarial worm *Onchocerca volvulus*, causing onchocerciasis (river blindness) in sub-Saharan Africa, some foci in Central and South America, and Yemen in the south of the Arabian Peninsula in Western Asia [16,17].

Substances contained in blackfly saliva and introduced by females during ingestion of blood from vertebrates cause simuliosis (simuliotoxicosis) [18,19], which may exert the most dramatic effect, i.e., anaphylactic shock leading to death in humans [20,21] and animals [22,23,24].

Despite the high prevalence of blackflies in some regions and a number of reported cases of human infestations, there is still little information about local and systemic reactions induced by these ectoparasites in various parts of the world, including Europe. The data on the epidemiology of dermatitis caused by blackflies are equally unsatisfactory.

In this study, we focused on the threats posed to human health by blackflies in southeastern Poland and on local signs and systemic reactions induced by the bites of these insects in humans.

## 2. Material and Methods

### 2.1. Study Area

The investigations were carried out in 2 attractive tourist regions located near the border with Ukraine and Belarus, i.e., Roztocze in the south-eastern part and Polesie in the central-eastern part of Lublin Province. Roztocze is situated between the basins of the Bug and Wieprz Rivers (in the north) and the San and Dniester Rivers (in the south). The numerous rivers in this area have the characteristics of rapidly flowing mountain streams. Polesie Lubelskie covers the basin of two rivers: the Wieprz and the Bug, with numerous tributaries and karst lakes.

### 2.2. Patients

The prospective study was conducted at 4 medical centers located in Lublin Province during the period of seasonal activity of blackflies between April and September in 2003–2005. The study involved 418 patients (256 females and 162 males) reporting blackfly attacks. The data on the patients (age and sex as well as the location and type of the skin lesions) were obtained from medical history and examination performed in the medical centers.

All patients underwent a general medical examination with assessment of blackfly toxin-induced skin lesions and other accompanying symptoms. During follow-up visits, the course of the disease was monitored until the local lesions and systemic reactions induced by the blackfly bites disappeared. The observations lasted from 1 to 30 days after the bites, depending on the persistence of the various disease signs in individual patients. The persistence of various disease symptoms in the patients was assessed.

The authors certify that they obtained all appropriate patient consent forms. The patients gave written consent for publication of clinical information and photographs. Institutional approval was not required for this case study.

### 2.3. Statistical Analysis

Quantitative variables were described as the mean, standard deviation, and minimum and maximum values. Since the data did not exhibit a normal distribution, the non-parametric Kolmogorov–Smirnov test was used for comparison of the distributions of one-dimensional statistical variables. The results of the analysis were considered statistically significant at the significance level of *p* ≤ 0.05.

The research material was analyzed statistically using Statistica 6.0. (Statistic for Windows, Statsoft, Palo Alto, CA, USA).

## 3. Results

At the site of the blackfly bite, there was itching in 94.74%, i.e., 245 females (95.70% among females) and 151 males (93.2% among males); burning in 55.02%, i.e., 153 females (59.76%) and 77 males (47.53%); edema in 44.02%, i.e., 116 females (45.31%) and 68 males (41.97%); erythema in 40.91%, i.e., 104 females (40.62%) and 66 males (40.74%); and hot skin in 22.49%, i.e., 56 females (21.87%) and 37 males (22.83%). A total of 370 subjects (88.52%) had only skin lesions at the site of the blackfly bites. A smaller percentage of the patients had other signs (Table 1). The skin lesions persisted from 1 to 30 days (mean 3.84 ± 2.99) in females and from 1 to 21 days (mean 3.68 ± 2.21) in males.

In 48 subjects, the skin lesions induced by blackfly bites were accompanied by systemic reactions. They were manifested by headache (6.22%), increased body temperature (2.87%), and arthralgia (1.20%). Lymphadenopathy was diagnosed in 2.63% of the patients, and menstrual disorders occurred in 1.91% of the examined patients (Table 1). 

**Figure 1:**
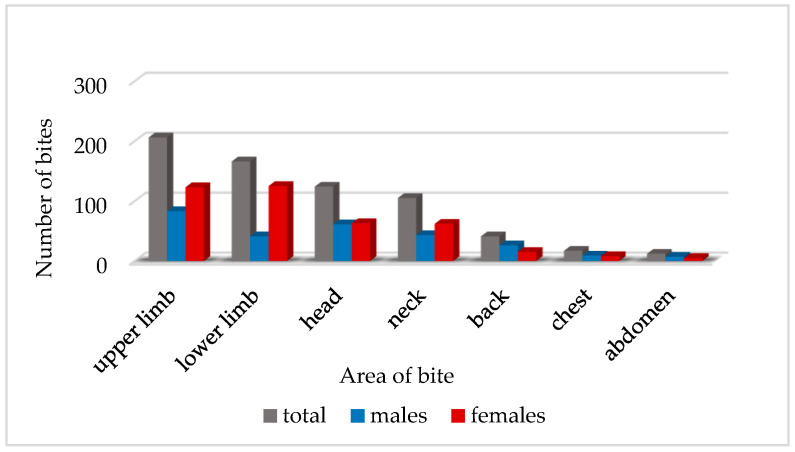
Number of blackfly bites on various parts of the patient’s body (*n* = 671) in the group of females (*n* = 401) and males (*n* = 270); *n*—number of all blackfly bites noted in the study.

**Figure 2:**
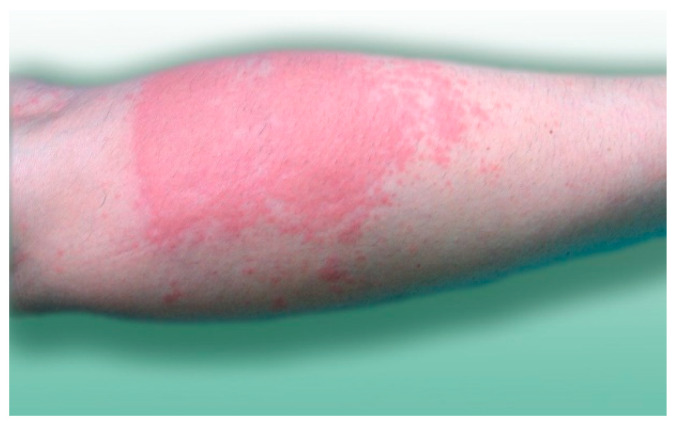
A wide urticarial plaque with small grouped papules as satellite lesions on the lower limb in a 50-year-old woman after blackfly bites.

**Figure 3:**
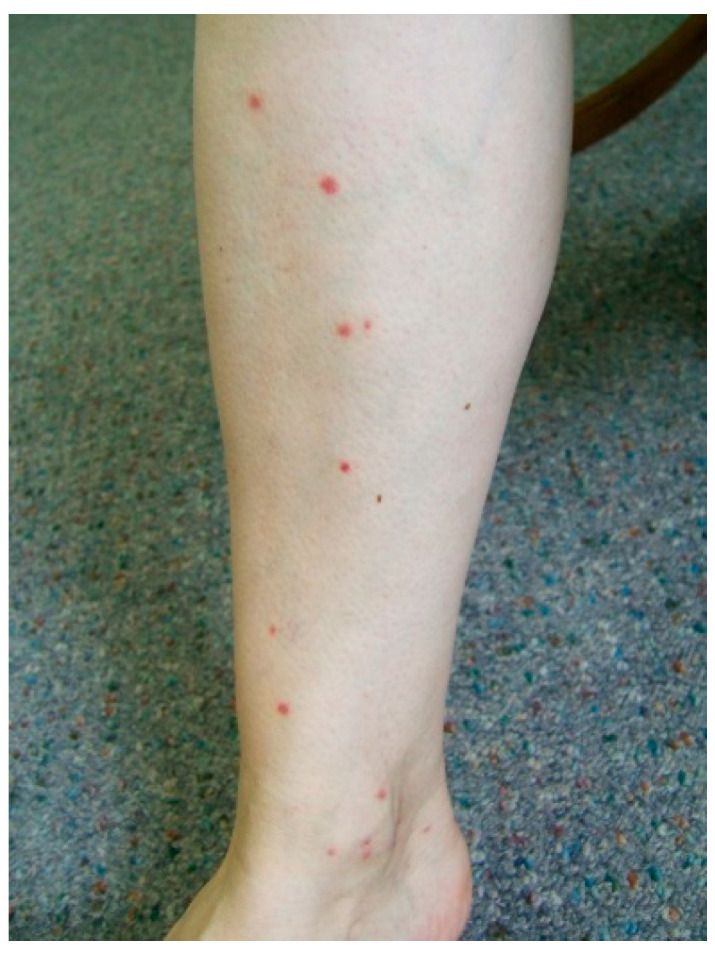
A few red papules on the lower limb of a 54-year-old woman after the blackfly bites.

The blackfly bites were most typically located in the area of the upper limb (48.8%), lower limb (39.71%), head (29.67%), and neck (25.12%), and less frequently on the back (9.81%), chest (4.07%), and abdomen (2.87%) (Figure 1, Figure 2 and Figure 3).

The patients were usually attacked by a large number of blackflies simultaneously. In total, 313 bites were detected on upper limbs. A lower number of blackfly bites (248) were noted on various parts of the lower limbs of the patients (Table 2).

The female patients were most often bitten by blackflies on the lower limbs (48.83%) and upper limbs (48.04%), but twice less frequently on the head (24.61%) and neck (24.22%). The lowest number of bites was detected on their back (5.86%), chest (3.13%), and abdomen (1.95%). In turn, the males were most often attacked by blackflies on the upper limbs (51.23%), head (37.65%), neck (26.54%), and lower limbs (25.31%). Bites on the back (16.05%), chest (5.56%), and abdomen (4.32%) were rarely detected in the male patients.

A total of 121 (28.94%) of the patients were attacked by blackflies once, and 297 (71.06%) of the patients were infested repeatedly. The skin lesions in the patients persisted from 1 to 10 days (mean 3.52 ± 1.89) after the first blackfly infestations and from 1 to 30 days (mean 3.90 ± 3.0) after the reinvasions. 

## 4. Discussion

In Europe, humans can be attacked outdoors by various bloodsucking insects, most often blackflies [5,25,26]; mosquitoes (Diptera: Culicidae) [27,28]; deer keds (Diptera: Hippoboscidae) [29,30,31]; and arachnids, e.g., ticks (Ixodida: Ixodidae) [32,33]. The most troublesome of these arthropods are the blackflies, which occur massively and attack hosts that are present in their habitats. The patients presented with numerous skin lesions at the insect bite sites on their bodies.

In the analyzed area, there were more cases of outpatient treatment of patients infested by blackflies than the bites by the castor bean tick *Ixodes ricinus*, which is widespread in southeastern Poland and transmits numerous tick-borne pathogens [32].

Female blackflies most often bite parts of the human body that are not protected by clothes, which was also observed in the present study. There were as many as 48.83% blackfly bites in the area of the lower extremities and 47.27% of cases of infestations of the upper limbs. In the most exposed parts of the body, blackflies most often attacked areas with good blood supply, i.e., the shin and foot on the lower limb and the forearm and hand on the upper limb.

Some of the differences in the location of the blackfly bites in the females and males can be mainly explained by the differences in the clothes worn by these groups. Women often wear clothes uncovering the lower limbs, as opposed to men, who usually expose the upper parts of the body during the holiday season. Hence, greater numbers of blackfly bites are noted on the upper limbs, head, neck, back, chest, and abdomen in males than in females. The different distribution of blackfly bites on female and male bodies may also be attributed to the different lifestyles in both groups (e.g., outdoor activity, recreation, and type of occupational work).

The small size of female blackflies (usually 1.5–4 mm in length) helps the insects to get under human clothes; hence, the bites and, consequently, the skin lesions caused by these insects were found on different parts of the patients’ bodies.

The substances contained in blackfly saliva induced inflammatory processes in the patients’ skin. The area, picture, and intensity of the skin lesions differed between the patients. This was probably associated with their specific physiological characteristics, which were not analyzed in this study. Swelling (44.02%) and erythema (40.91%) often developed in the area surrounding the bite site. In the majority of the blackfly bite cases (94.74%), the patients experienced itching as well as a burning sensation, hot skin, and pain.

Cases of human dermatitis caused by *Simulium* have also been reported by other authors from various European countries, e.g., [34,35,36,37,38,39,40,41,42,43] and other parts of the world, e.g., [44,45,46,47,48].

Bleeding from damaged blood vessels in humans may persist for a long time due to the presence of anti-hemostatic components in blackfly saliva [49,50,51,52,53,54,55]. Additionally, when scratched, swollen papules and vesicles filled with serous fluid, serous/blood fluid, or blood may bleed for a long time and heal slowly [39,56]. Petechiae may form within the erythema as well [56,57,58]. The hemorrhages and severe inflammatory reactions observed in the present study and by other authors reporting on blackfly infestation cases are the result of extensive damage to the skin and blood vessels caused by the mouth organs of blackflies and the immunomodulatory effect of the components of their saliva [46,51,52,54,55,59,60,61,62,63].

Systemic reactions to the blackfly bites were observed in the patients eight times less frequently than skin lesions. The most common systemic reactions detected in the present study and described in the literature include headache, abdominal pain, arthralgia, nausea, weakness, increased body temperature, chills, menstrual disorders, nervous agitation, and cardiovascular disorders (e.g., [41,56,58,64,65]).

Toxins contained in blackfly saliva can trigger anaphylaxis and allergic reactions of varying severity, e.g., urticaria, Quincke’s edema, bronchospasm, hypotension, and anaphylactic shock [21,58,61,65,66]. 

The results of the present study and descriptions of symptoms in patients attacked by blackflies in other regions show the diversity of effects on humans infested by these ectoparasites, which should be considered in the differential diagnosis of diseases caused by bloodsucking arthropods.

## 5. Conclusions

Blackflies (*Simulium* spp.) occur commonly in southeastern Poland and pose a considerable threat to the health of its inhabitants. The massive occurrence of blackflies and their high aggressiveness towards hosts manifested by the substantial number of attacks of humans engaged in recreational and occupational activities suggest that, in some regions, blackflies should be regarded as an important causative factor of dermatitis, which may also be accompanied by systemic reactions.

Patients attacked by blackflies most often present with skin lesions characterized by varied severity and appearance, usually small papules and popular and purpuric lesions. In turn, systemic reactions (mainly headache, fever, and lymphadenopathy) to the toxic components of blackfly saliva injected by these insects during blood ingestion are observed less frequently.

## Figures and Tables

**Table 1:** Symptoms of skin lesions and systemic reactions induced by blackfly infestations in patients from southeastern Poland (*n* = 418).

Skin Lesions and Systemic Reactions	Cases	
	*n*	%
Skin lesions	418	100
Pruritus	396	94.74
Burning	230	55.02
Edema	184	44.02
Erythema	171	40.91
Hot skin	94	22.49
Pain	56	13.40
Tingling	20	4.78
Other	7	1.67
Systemic reactions	48	11.48
Headache	26	6.22
Fever	12	2.87
Lymphadenopathy	11	2.63
Menstrual disorders	8	1.91
Arthralgia	5	1.20
Other	1	0.24

*n*: number of patients.

**Table 2:** Localization of blackfly bites in the patients (*n* = 671).

Body Area	Number of Bites	
	*n*	%
Lower limb:	248	100
Shin	113	45.56
Foot	60	24.19
Thigh	36	14.52
Knee	29	11.69
Groin	10	4.04
Upper limb:	313	100
Forearm	135	43.13
Hand	119	38.02
Elbow area	24	7.67
Arm	23	7.35
Armpit	12	3.83
Head	129	100
Frontal and buccal areas	68	52.71
Orbital area	17	13.22
Temporal area	35	27.09
Labial area	2	1.55
Parietal and occipital areas	7	5.43

*n*: number of bites noted in the study.

## Data Availability

The data presented in this study are available on request from the corresponding author. The data are not publicly available due to patient privacy.
